# Supplementary material for: Identification and Evaluation of Hub Long Noncoding RNAs and mRNAs in High Fat Diet Induced Liver Steatosis
Source: Nutrients. 2023 Feb 14;15(4):948. doi: 10.3390/nu15040948 (PMC9963248; doi:10.3390/nu15040948)
Supplement: Supplementary file 1 [file nutrients-15-00948-s001.zip › nutrients-2174833-supplementary.pdf]

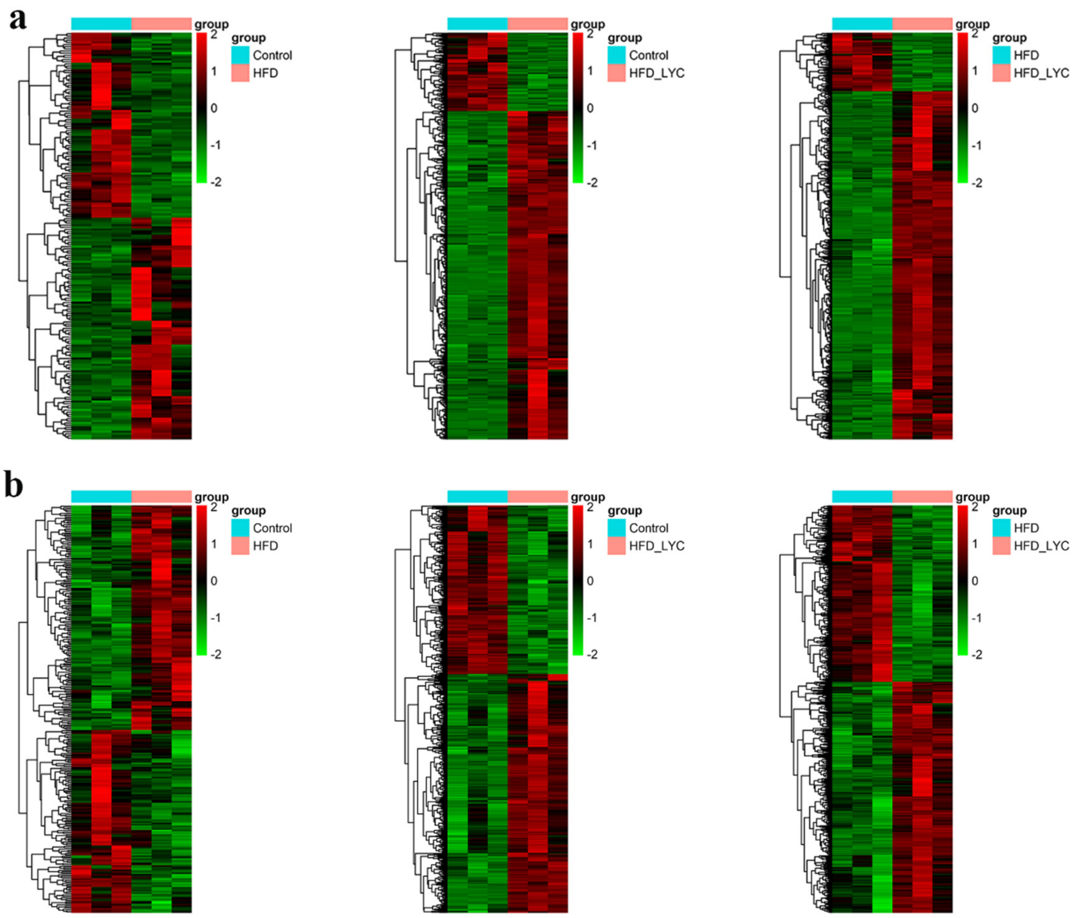

Figure S1. Identification of differentially expressed lncRNAs and mRNAs among control, HFD, HFD-LYC group. (a) lncRNAs; (b) mRNAs. HFD, high fat diet; HFD-LYC, high fat diet +lycopene.

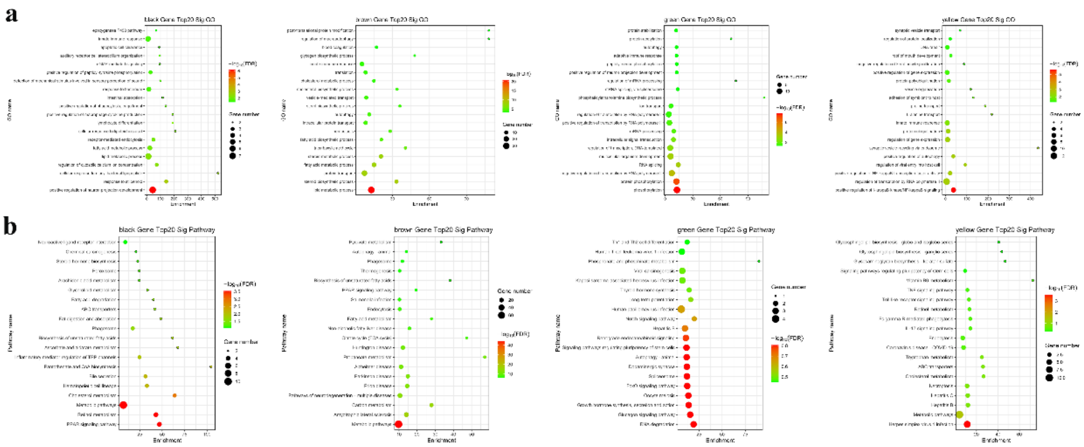

Figure S2. Functional enrichment analysis in the brown, yellow, green and black modules. (a) Enriched GO analysis of mRNAs; (b) Enriched KEGG pathway analysis of mRNAs.

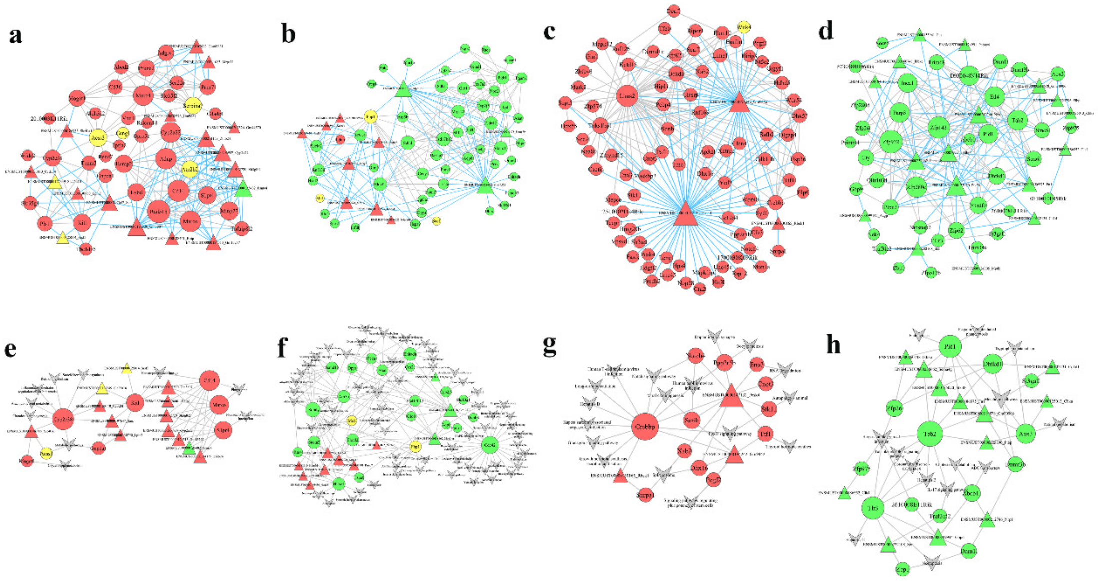

Figure S3. Module lncRNA–mRNA net of hub genes in the brown (a), yellow (b), green (c) and black (d) modules. Module lncRNA–mRNA pathway net of hub genes in the brown (e), yellow (f), green (g) and black (h) modules. Circles represent mRNAs, triangles represent lncRNAs, and gray polygons repre-sent pathways. Red represents upregulation, green represents down-regulation, yellow represents both up- and down-regulation (because pairwise comparisons are three groups). The size of the graphs represents the level of intramodular connectivity of hub genes in the network.

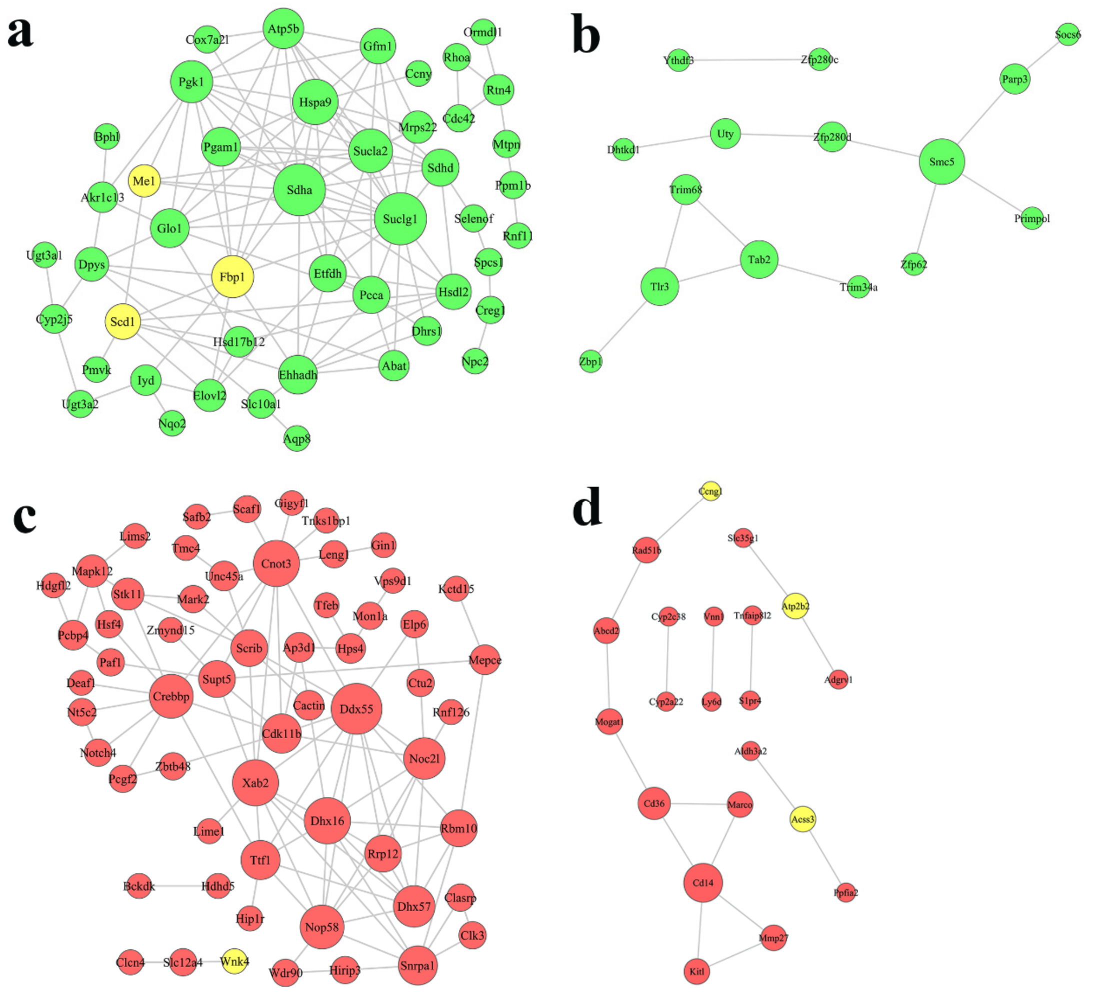

Figure S4. Construction of protein-protein interaction (PPI) networks in the brown (a), yellow (b), green (c) and black (d) modules.
